# Prostate tumor attenuation in the nu/nu murine model due to anti-sarcosine antibodies in folate-targeted liposomes

**DOI:** 10.1038/srep33379

**Published:** 2016-09-20

**Authors:** Zbynek Heger, Hana Polanska, Miguel Angel Merlos Rodrigo, Roman Guran, Pavel Kulich, Pavel Kopel, Michal Masarik, Tomas Eckschlager, Marie Stiborova, Rene Kizek, Vojtech Adam

**Affiliations:** 1 Central European Institute of Technology, Brno University of Technology, Purkynova 123, CZ-612 00 Brno, Czech Republic; 2 Department of Chemistry and Biochemistry, Mendel University in Brno, Zemedelska 1, CZ 613 00 Brno, Czech Republic; 3 Department of Pathological Physiology, Faculty of Medicine, Masaryk University, Kamenice 5, Brno CZ-625 00, Czech Republic; 4 Department of Chemistry and Toxicology, Veterinary Research Institute, Hudcova 296/70, CZ 621 00 Brno, Czech Republic; 5 Department of Pediatric Hematology and Oncology, 2nd Faculty of Medicine, Charles University, and University Hospital Motol, V Uvalu 84, CZ-150 06 Prague 5, Czech Republic; 6 Department of Biochemistry, Faculty of Science, Charles University, Albertov 2030, CZ-128 40 Prague 2, Czech Republic

## Abstract

Herein, we describe the preparation of liposomes with folate-targeting properties for the encapsulation of anti-sarcosine antibodies (antisarAbs@LIP) and sarcosine (sar@LIP). The competitive inhibitory effects of exogenously added folic acid supported the role of folate targeting in liposome internalization. We examined the effects of repeated administration on mice PC-3 xenografts. Sar@LIP treatment significantly increased tumor volume and weight compared to controls treated with empty liposomes. Moreover, antisarAbs@LIP administration exhibited a mild antitumor effect. We also identified differences in gene expression patterns post-treatment. Furthermore, Sar@LIP treatment resulted in decreased amounts of tumor zinc ions and total metallothioneins. Examination of the spatial distribution across the tumor sections revealed a sarcosine-related decline of the MT1X isoform within the marginal regions but an elevation after antisarAbs@LIP administration. Our exploratory results demonstrate the importance of sarcosine as an oncometabolite in PCa. Moreover, we have shown that sarcosine can be a potential target for anticancer strategies in management of PCa.

Prostate cancer (PCa) was the most common cause of cancer in men in the USA in 2013, afflicting one in nine men over the age of 65^1^. Distinct sets of genes, proteins, and metabolites orchestrate cancer progression from a precursor lesion to localized disease and finally to metastatic cancer^2^. However, despite a broad knowledge of gene/protein expression profiles in PCa, complex metabolomic alterations are still not yet understood. PCa-specific metabolites can be effective targets that are exploitable in not only early diagnostics but also in the enhancement of therapeutic possibilities.

In 2009, Sreekumar and coworkers discovered that the urinary level of sarcosine (*N*-methylglycine), an intermediate by-product of glycine synthesis and degradation, increased within the localized PCa and metastatic PCa cohorts^3^. Although the linkage of sarcosine with PCa development^4^,^5^,^6^ and its potential in the diagnosis of early-stage tumors have been reported^7^, its use as a biomarker is still unclear^8^. Khan *et al.* demonstrated that addition of sarcosine induced invasion and intravasation in an *in vivo* PCa model^9^. Their comprehensive study of sarcosine-related enzymes shed light on sarcosine as a substantial PCa oncometabolite that can impact the oncogenic potential of prostate cells. Similarly, our *in vitro* pilot study revealed that sarcosine supplementation affects the redox status of PC-3 cells^10^. Hence, we used our anti-sarcosine antibodies (antisarAbs), whose immunoperformance was tested in ref. 11, as cargo for liposomal nanotransporters (antisarAbs@LIP) to promote the internalization of these antibodies into intracellular regions and the subsequent neutralization of sarcosine. To increase the liposomal shuttling efficiency, the outer surface was modified with folic acid (FA)^12^. This Trojan horse process performed through FA-folate receptor affinity promotes cellular uptake *via* clathrin-mediated endocytosis, which enables the passage of larger objects (including liposomes) across cellular membranes^13^.

The main objective of our study was to determine whether the administration of antisarAbs and sarcosine liposomal formulations affected PC-3 xenografts in terms of tumor growth and gene expression patterns to obtain insight into the uncertain role of sarcosine in PCa development. To enhance the uptake into prostate tumor tissue, we utilized FA-targeted liposomes, which enabled us to investigate sarcosine-related cellular issues in highly specific way. A special emphasis was placed on the administration effects on a key regulatory factor in the intermediary metabolism of prostate cells (zinc) and a substantial transporter protein involved in zinc homeostasis (metallothioneins, MTs).

## Results

### Characterization and *in vitro* intracellular uptake of 1,2-dioleoyl-sn-glycero-3-phosphoethanolamine (DOPE) liposomes

Liposomal sarcosine and antisarAbs were produced using the lipidic thin-film hydration method^14^ as well as control liposomes without cargo (hereinafter denoted as No cargo). Subsequent surface modification with FA was achieved through an esterification reaction using the zero-length linker 1,1′-carbonyldiimidazole (CDI). Table 1 demonstrates the relatively high yields of encapsulation for both types of cargos [loading efficiency (LE) 78.9% for sarcosine, LE 56.2% for antisarAbs].

**Figure 1:**
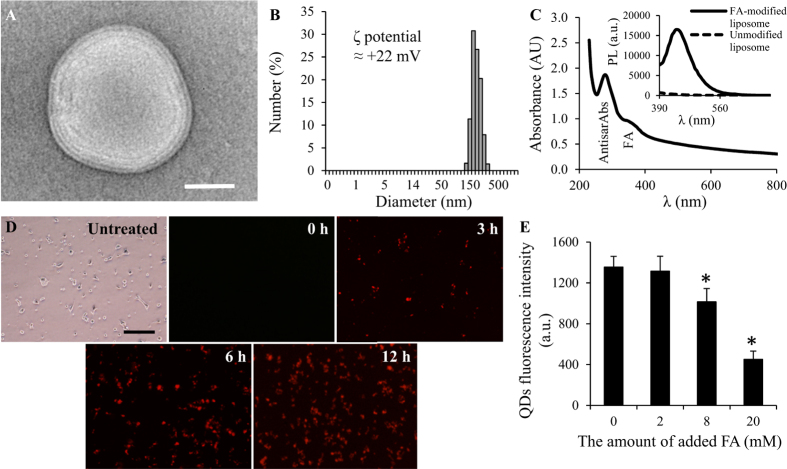
Characterization of FA-modified liposomal formulations of sarcosine and antisarAbs. (**A**) TEM micrograph (length of scale bar is 50 nm) and (**B**) dynamic light scattering analyzed in PBS, pH 7.4, with corresponding ζ potential inserted. (**C**) UV-Vis absorption spectrum of antisarAbs-bearing liposomes (λ = 270 nm corresponds to encapsulated antisarAbs, and λ = 365 nm corresponds to surface modification with FA) with the inserted photoluminescence spectra of liposomes before and after modification with FA (λ_exc_ = 360 nm). (**D**) Time dependence of the cellular uptake of QD-labeled antisarAbs@LIP into PC-3 cells obtained via inverted fluorescence microscopy (length of scale bar is 200 μm). Cells were incubated with 10 μM QD-labeled antisarAbs@LIP. (**E**) Competitive inhibitory effects in PC-3 cells. Values represent the mean ± SD of three experiments. Asterisks indicate significant differences (*p* < 0.05).

Transmission electron microscopy (TEM) observations revealed spherical multilamellar vesicles (Fig. 1A) averaging 172 nm in diameter (Fig. 1B). FA-modified liposomes exhibited ζ potential ≈ +22 mV. Successful surface modification was confirmed using optical methods (Fig. 1C). The absorption spectra of antisarAbs@LIP revealed two absorption maxima, one due to the presence of encapsulated antibodies (λ = 280 nm) and one due to FA (λ = 365 nm). Liposome suspensions exhibited the characteristic fluorescence emission peaks of FA (insert in Fig. 1C), suggesting successful coating of FA molecules onto the liposome surfaces.

*In vitro* cellular uptake experiments were performed using human prostate cancer PC-3 cells, which overexpress folate receptors^15^,^16^. The uptake of quantum dots (QD)-labeled antisarAbs@LIP was evaluated over time (0–12 h) and is shown in Fig. 1D. After 6 h of incubation, the fluorescence considerably increased, and high uptake was also measured after 12 h of incubation, demonstrating that liposomes interacted with PC-3 cells. The uptake of substances into cells *via* folate receptors is reduced in the presence of excess amounts of FA due to competitive inhibitory effects. Hence, to ascertain that liposomes were indeed internalized through folate targeting, we assessed the cellular uptake of QD-labeled antisarAbs@LIP in the presence of FA, and the results from this assay are illustrated in Fig. 1E. The cellular uptake of QD-labeled antisarAbs@LIP was significantly reduced in PC-3 cells as the FA concentration increased. Thus, our results indicate the important role of folate receptors in the cellular uptake of our liposomal formulations in PC-3 cancer cells.

### *In vivo* effects of antisarAbs@LIP and sar@LIP on PC-3 xenografts

**Figure 2:**
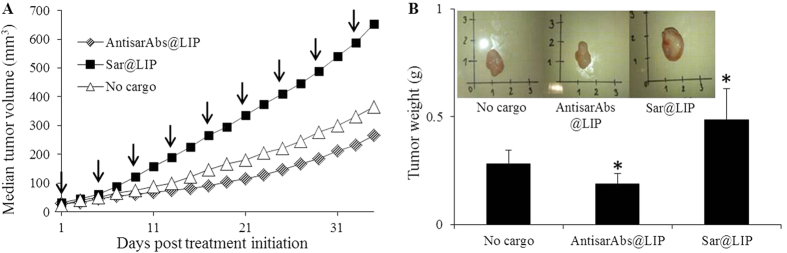
*In vivo* antitumor efficacy of liposomal formulations in PC-3 tumor xenografts. (**A**) Average tumor volume in nude mice bearing subcutaneous PC-3 tumors. Arrows indicate the time of therapeutic injections (*i*.*p*.). (**B**) Average tumor weight at the endpoint of the experiment. Inserted are chosen photographs of PC-3 tumors after termination. Values are expressed as the mean of five independent replicates (*n* = 5). Vertical bars indicate standard error. Asterisks indicate significant differences (*p* < 0.05) compared to the group treated with empty liposomes.

Treatment of mice PC-3 xenografts with various formulations of liposomes began when the tumor volume reached approximately 30 mm^3^ and was considered the first treatment day. The mice in all experimental groups were administered treatments nine times by intraperitoneal injection for 35 days. The tumor growth time profile obtained by measuring the tumor diameter in the test animals twice per week is presented in Fig. 2A. Treatment with sar@LIP remarkably enhanced tumor growth from approximately the 7^th^ day of administration. In contrast, antisarAbs@LIP administration showed moderate antitumor activity. Tumor weight was calculated to evaluate the impact on tumors and is shown in Fig. 2B. Administration of antisarAbs@LIP led to the inhibition of tumor weight by 32.5%. Notably, the mean weight of the excised tumors was approximately 71.4% higher in mice treated with sar@LIP than in mice treated with empty liposomes (No cargo). No mice died or had to be euthanized before the endpoint of the experiment, and administration did not cause significant differences in mouse weight.

### Gene expression patterns and apoptosis in excised tumors

**Figure 3:**
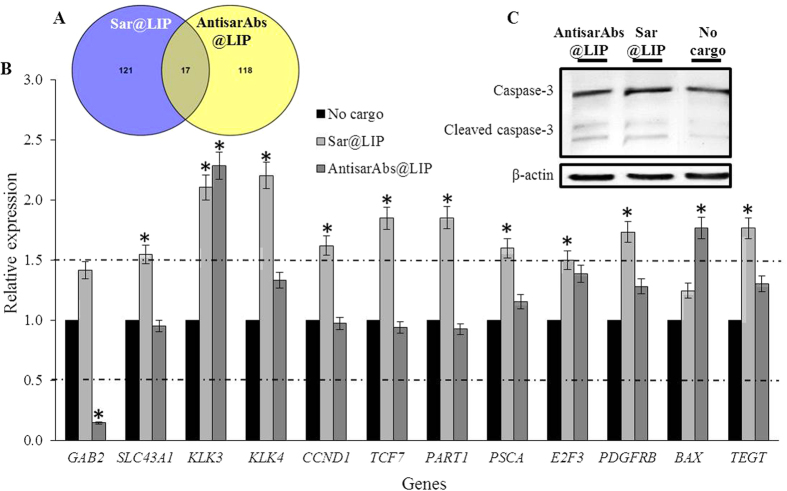
Effects of administration on the gene expression patterns. (**A**) Venn diagram showing the overlapping genes that were up-regulated after both types of administration. (**B**) Representation of the relative expression levels of genes specific to any aspect of PC, which were significantly up- or down-regulated in post-treatment tumor tissues. *GAB2* – *Growth factor receptor-bound protein 2 (GRB2*)*-associated binding protein 2*; *SLC43A1* – *Solute carrier family 43*, *member 1*; *KLK3* – *Kallikrein 3 (prostate-specific antigen*) *PSA*; *KLK4* – *Kallikrein 4 (prostase*, *enamel matrix*, *prostate*); *CCND1* – *Cyclin D1*, *alpha polypeptide*; *TCF7* – *Transcription factor 7 (T-cell specific*, *HMG-box*); *PART1* – *Prostate androgen regulated transcript 1*; *PSCA* – *Prostate stem cell antigen*; *E2F3* – *E2F transcription factor 3*; *PDGFRB* – *Platelet-derived growth factor receptor*, *beta polypeptide*; *BAX* – *Bcl2-associated X protein*, *TEGT* – *Transmembrane BAX inhibitor motif containing 6*. Values are expressed as the mean of three independent replicates (*n* = 3). Vertical bars indicate standard error. Asterisks indicate significant differences (*p* < 0.05) compared to the group treated with empty liposomes. (**C**) Western blot analysis for the xenografts expression of caspase-3 and cleaved caspase-3.

To understand the gene expression patterns, we performed electrochemical microarray analyses of RNA isolated from tumor tissues. We compared the effects of both administration types with the expression patterns of PC-3 xenografts treated with empty liposomes (complete array heatmaps are depicted in Supplementary Figure 1, together with a control expression heatmap of benign prostate PNT1A cells). The Venn diagram in Fig. 3A illustrates that the administration of sar@LIP and antisarAbs@LIP resulted in significant differential expression patterns, with only 17 overlapping genes up-regulated as a result of treatment with either sar@LIP or antisarAbs@LIP. The other (*n* = 121 and *n* = 118, respectively) up-regulated genes were uniquely connected to individual treatments. The complete lists of up- and down-regulated genes and their relative expression levels are shown in Supplementary Tables 2 and 3. Furthermore, we highlighted 12 genes that were specific to any aspect of PCa (Fig. 3B). Most (*n* = 10) were significantly up-regulated by the presence of exogenously added liposomal sarcosine (*p* < 0.05). In contrast, antisarAbs@LIP administration led to the up-regulation of only two of these genes (*KLK3* [*kallikrein 3*] and pro-apoptotic *BAX* [*Bcl2-associated X protein*) and, moreover, caused significant down-regulation of *GAB2 (growth factor receptor-bound protein 2 (GRB2*)*-associated binding protein 2*). The annotation analysis from GoMiner indicated that some of those genes, including *CCND1 (cyclin D1*, *alpha polypeptide*), *BAX*, *TEGT (transmembrane BAX inhibitor motif containing 6*), *E2F3 (E2F transcription factor 3*) or *PDGFRB (platelet-derived growth factor receptor*, *beta polypeptide*), were related to the positive regulation of cell proliferation and apoptosis. Noteworthy, sar@LIP induced the up-regulation of *SLC43A1 (solute carrier family 43*, *member 1*), whose major role is amino acid transport and the supply of nutrients into cancer cells. We further detected the apoptosis-related proteins in excised xenografts. Figure 3C demonstrates that antisarAbs@LIP treatment slightly increased the amount of cleaved caspase-3 when compared to sar@LIP administration, which is in agreement with the expression of pro-apoptotic *BAX*.

### Zinc and MT content in tumor tissue

**Figure 4:**
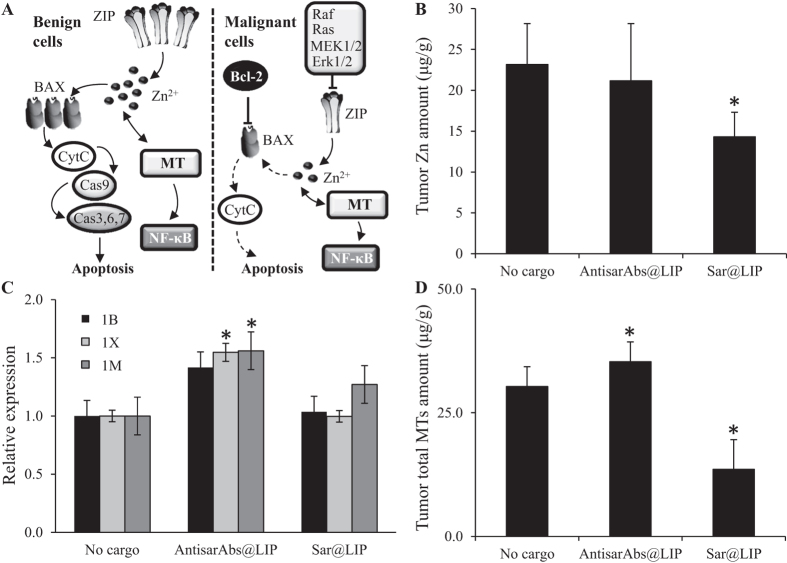
Effect on treatment on tumor zinc and metallothionein content. (**A**) Schematic depiction of zinc and metallothioneins and their importance in benign and malignant prostate cells. In malignant cells, ZIP transporters are inhibited through the Ras-Raf-Mek-Erk cascade, resulting in a decrease of Zn^2+^ ions in the cytosol. This phenomenon, together with Bcl-2 activity, inhibits the pro-apoptotic activity of cytochrome 1 *c* (CytC) in these cells. MT expression is closely linked to the Zn amount in the intracellular space. (**B**) Zinc levels in PC- 3 tumors after administration of liposomal sarcosine and antisarAbs. (**C**) Relative expression levels of mRNAs encoding different human metallothionein isoforms (1B, 1X and 1M) in tumor tissue after administration of liposomal sarcosine and antisarAbs, analyzed using electrochemical microarray. (**D**) Determination of total MTs using the Brdicka reaction after sample denaturation. Values are expressed as the mean of three independent replicates (*n* = 3). Vertical bars indicate standard error. Asterisks indicate significant differences (*p* < 0.05) compared to the group treated with empty liposomes.

Zinc homeostasis maintains physiological conditions in PCa, and zinc levels are significantly diminished in cancerous tissue (Fig. 4A). Thus, we determined the zinc levels in excised tumors. Figure 4B illustrates that sar@LIP administration significantly decreased the amount of zinc in tumor tissue (mean of 14.3 μg/g compared to 23.2 μg/g in mice treated with empty liposomes). In contrast, treatment with antisarAbs@LIP did not alter zinc levels in tumor tissue. Zn regulation is partially performed through MT proteins. Thus, we focused on the expression profiles of mRNAs encoding three isoforms of MT (1B, 1X and M). Figure 4C indicates that only antisarAbs@LIP administration significantly up-regulated MT1X and MTM mRNA expression levels (*p* < 0.05). To further elucidate the disparities in MT levels in tumor tissue after the treatments, we performed electrochemical examination by differential pulse voltammetry utilizing Brdicka’s solution as an electrolyte. Because the MTs exhibit exceptional thermal stability, tumor homogenates were denatured and filtered to remove undesired interfering particles. Figure 4D demonstrates that voltammetry partially supported the results obtained using microarray and that antisarAbs@LIP administration elevated the MT content to 35.3 μg/g compared to mice treated with empty liposomes (mean 30.3 μg/g). In contrast, sar@LIP treatment significantly decreased MT content in xenograft tumors (mean 13.6 μg/g). Overall, our results indicate a plausible linkage between sarcosine metabolism and metal homeostasis in PCa.

### Examination of tumor cross-sections

**Figure 5:**
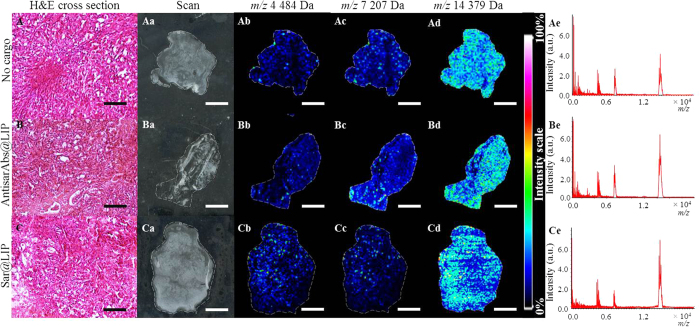
Administration results in disparities in a spatial distribution of proteins. H&E-stained cross sections of PC-3 xenografts (**A**) without treatment and after treatment with (**B**) liposomal antisarAbs and (**C**) liposomal sarcosine. Tumor sections (scale bar = 100 μm) were (a) scanned and utilized for MALDI-IMS profiling. Normalized pseudo-colored images of the spatial distribution of proteins with *m/z* values of: (b) 4484 ± 0.25% Da, (c) 7207 ± 0.25% Da and (d) 14379 ± 0.25% Da. (e) Mean mass spectrum of spectra obtained from MALDI IMS of PC-3 tumors. DHB was used as a matrix. The conditions used to acquire the MALDI spectra were as follows: 1,600 shots per raster spot, 65% laser energy and spatial resolution of 100 μm. All treated animals were euthanized 35 days after dosing. The scale bar length for the MALDI-IMS scans is 2 mm.

We further visualized molecules using imaging mass spectrometry (IMS). Figure 5 shows the images that were first obtained by performing matrix-assisted laser/desorption ionization (MALDI)-IMS and a hematoxylin and eosin (H&E) staining of the tumor section. The tumor specimens stained by H&E were ascertained by microscopic examination, which revealed no observable histological changes in the control and post-treatment excised tumor xenografts (Fig. 5A–C). Figure 5Aa–Cd depicts typical images that were obtained from MALDI-IMS and were used to visualize the distribution of predominant peaks (*m/z* 4484 Da, *m/z* 7207 Da and *m/z* 14739 Da) determined in the mean mass spectra represented in Fig. 5Ae–Ce. Generally, our treatment protocol did not result in expression pattern changes. However, our treatment protocol did affect the intensities of chosen peaks. The highest differences were found in the case of *m/z* 7207 Da after sar@LIP administration, with a substantially reduced ion signal compared to empty liposomes and antisarAbs@LIP. The spatial distribution was relatively homogenous, except for the margins of tumors treated with sar@LIP, where the ion signal was nearly lost. AntisarAbs@LIP treatment resulted in elevated signal at the margins. Subsequent on-tissue digestion resulted in the successful identification of three predominant peaks as follows: *m/z* 4 484 Da corresponded to beta-defensin 1 (DEFB1, approx. MASCOT score 65.2), *m/z* 7 207 Da corresponded to MT isoform 1X (MT1X approx. score 55.0), and *m/z* 14 739 Da corresponded to cytochrome b5 (CYB5, approx. score 78.1).

## Discussion

In this study, we demonstrated that FA-modified liposomes are suitable to target cancer cells, as folate receptors are overexpressed in a wide variety of tumors, including PCa^17^. We provide evidence that sarcosine plays a substantial role in PCa progression and can be a potential target for the development of novel treatment modalities.

PCa remains a leading cause of illness and death among men in the US and Western Europe. Autopsy series have revealed small prostatic carcinomas in up to 29% of men age 30 to 40 years and 64% of men age 60 to 70 years. Moreover, the risk of PCa is 1 in 6, and the risk of death due to metastatic PCa is 1 in 30^18^. Despite these astounding statistics, the mechanism of prostate tumorigenesis is still not well understood, although it appears to be multifactorial.

Thus, the rationale of encapsulating sarcosine and antisarAbs is primarily to further understand the role of sarcosine as a suspicious oncometabolite important in PCa development. We used a liposomal transporter due to the inability of antibodies to enter the intracellular space, which can occur through the well-described FA-folate receptor interaction^19^,^20^,^21^, leading to folate receptor-mediated endocytosis. The encapsulation into the FA-modified liposomes produced stable delivery systems, which enabled highly specific investigation of sarcosine-induced cellular events. We anticipate that exploitation of advanced nanomaterials for research on cancer metabolism can benefit from their exceptional properties, including specificity, prolonged circulation and wide payload spectrum of studied substance. Indeed, our results confirmed interactions between PC-3 cells and our designated nanotransporter *in vitro*. These were first provided by electrostatics between the negatively charged proteoglycans contained on cellular surfaces and the positively charged liposome surface. Second, folate targeting facilitated the endocytosis of liposomes, as was proved by our competitive inhibitory assay (Fig. 1E). Considering the *in vivo* tumor microenvironment, the enhanced permeability and retention effects should be taken into account as important mediators of the increased liposome accumulation in the tumor mass^22^.

As approximations of human disease, animal models of cancer are critical for the understanding of the pivotal mechanisms of cellular transformation and the development of novel antitumor therapies. Therefore, it is important to have systems that can mimic the tumor biology observed in patients, providing accurate information on the signaling networks and biochemical pathways in the tumor microenvironment^23^. The direct addition of sarcosine imparted an invasive phenotype to benign prostate cells, and the number of motile prostate cells was significantly higher upon sarcosine treatment^3^. Therefore, in this work, PC-3 cells were grown as xenografts in nude mice as a model for sar@LIP and antisarAbs@LIP administration. We found that sar@LIP exerted a dramatic tumor growth-supporting activity, leading to increases in tumor volume and weight. In contrast, antisarAbs@LIP exerted mild antitumor activity, as shown in Fig. 2A,B.

We acknowledge that these findings are in agreement with results published by Khan and coworkers, who reported the substantial role of sarcosine in invasion and intravasation in an *in vivo* PCa model^9^. We believe that these findings portray the importance of sarcosine in PCa progression. Furthermore, we expanded this knowledge to include the possible exploitation of anti-sarcosine antibodies, which caused mild antitumor effects. However, further examination must be performed to distinguish and overcome possible drawbacks, such as their low tissue penetration^24^.

Sarcosine is produced from glycine by the enzymatic action of glycine-*N*-methyltransferase (GNMT), which is frequently elevated in localized and metastatic PCa relative to benign tissue^25^. However, how the elevated sarcosine levels affect the cellular microenvironment is uncertain. As a first level of organization, we examined the administration-related gene expression patterns of tumors using microarrays, which provided a snapshot of the cell functions and processes of thousands of genes and should yield insights concerning changes in gene expression associated with tumor cellular dysfunction and any concomitant regulatory pathways. Gene microarrays revealed the interesting differential expression profiles of a number of cancer-related genes in post-administration analyses. In particular, the *c-JUN* and *c-FOS* proto-oncogenes, *MMP2*, an independent predictor of decreased PCa survival rates, and *CCDN1*, a cell cycle progression regulator^26^,^27^,^28^, were up-regulated only after sar@LIP treatment, illustrating that sarcosine can markedly affect the cellular microenvironment of PCa cells. Within the significantly regulated genes, we further selected those that are associated with any aspect of PCa (Fig. 3B). Noteworthy, *GAB2*, which encodes the GAB2 scaffolding protein, which serves as a platform for the assembly of signaling systems fundamental for PCa development^29^, was significantly down-regulated by antisarAbs@LIP (*p* < 0.05). This prevents PCa cells from interactions with signaling molecules, which are vital for tumorigenesis^30^. On the contrary, sar@LIP treatment induced slight up-regulation of *GAB2*, which supports its tumor stimulatory role. These findings describe a plausible mechanism for the moderate antitumor activity of antisarAbs@LIP, together with the capability to up-regulate *BAX*, encoding Bcl-2-like protein 4, which accelerates programmed cell death through increase of the opening of the mitochondrial voltage-dependent anion channels, which leads to the loss in membrane potential and the release of cytochrome c (Fig. 4A)^31^. In contrast, sar@LIP induced the up-regulation of a broad spectrum of genes related to the positive induction of cell proliferation (*CCND1*, *E2F3*, *PDGFRB* and *TEGT*, a described *BAX* inhibitor), which corresponds to the tumor growth enhancement observed *in vivo* after sar@LIP administration. Interestingly, both types of treatments triggered up-regulation of *KLK3*, which encodes prostate-specific antigen (PSA) - a serine protease produced by epithelial cells in prostate, which represents the gold standard biomarker of PCa^32^. We hypothesize that this phenomenon is due to utilization of FA for liposomes surface modification. Tisman and Garcia have demonstrated that FA supplementation is associated with a serum PSA acceleration, while its withdrawal from diet resulted in a significant PSA decline^33^. Despite that there is a lack of information describing a stimulation of PSA through FA exploited for folate targeting. Hence, further research is needed to rule out this phenomenon. Proliferative stimuli are highly dependent on metabolic responses. A critical aspect of the re-programming of cancer cell metabolism involves changes in the glycolytic pathway, referred to as the Warburg effect. Glutaminase is a key enzyme involved in the Warburg effect, and its role is to convert glutamine, an amino acid essential for tumor growth, to glutamate^34^. In sar@LIP, but not in antisarAbs@LIP-administered tumors, we found significantly up-regulated expression of *GLS*, which encodes glutaminase. Furthermore, PCa cells overexpress L-type amino acid transporters (LATs) to enhance their nutrient status and to stimulate cell growth^35^. Our results also illustrate that sar@LIP administration results in significant up-regulation of *SLC43A1*, which encodes LAT3 proteins. LAT3 can contribute to various signaling pathways, including mammalian target of rapamycin complex 1, implicated in tumorigenesis^36^. Our pioneering data highlight the important role of sarcosine in PCa cells. However, the mechanism of action by which sarcosine affects these genes needs to be further investigated.

In normal prostate tissue, Zn^2+^ ions enter cells *via* ZIP (Zrt- and Irt-like protein) transporters. Crossing the membranes, zinc induces BAX to form pores in the outer mitochondrial membrane, through which cytochrome c (CytC) is released into the cytosol. CytC then interacts with caspases to cause apoptosis (Fig. 4A). The growth effect of low intracellular zinc levels results from the elimination of the pro-apoptotic effects of zinc. Together with increased energy production, the growth effect promotes transformation into energy-efficient, proliferative, malignant cells. Our data suggest that sar@LIP administration is significantly associated with decreased amounts of zinc in tumor tissue (*p* < 0.05). We anticipate that the disruption of the tight homeostatic control of the cellular zinc level serves as an indicator of the stimulant effects of sarcosine on PCa cells. AntisarAbs@LIP treatment resulted in the partial regression of zinc homeostasis. Thus, sarcosine accumulation and zinc depletion appear to play important roles in PCa development.

MTs are intracellular proteins that bind transition metals, including zinc. Hasumi *et al.* demonstrated that similarly to zinc, decreased levels of MT are associated with PCa^37^. Furthermore, zinc treatment up-regulates MT expression. Notably, after antisarAbs@LIP administration, similar effects were achieved on both the mRNA (isoforms 1B; 1X and 1M) and protein levels (total MTs). In contrast, sar@LIP significantly decreased the amount of total MT protein but not the transcribed mRNA. These results correspond to our preliminary *in vitro* study with PC-3 cells administered sarcosine^10^. The amount of MT was reduced in sarcosine-supplemented cells in a time-dependent manner. Alterations in zinc-metallothionein metabolism are described as the early signs of PCa progression^38^, and our data reveal that sarcosine decreases their amounts in tumor tissue. MT expression is tightly regulated by the intracellular zinc pool through metal-regulatory transcription factor 1. Thus, MT down-regulation is likely linked to decreased zinc, which reflects the proliferative and malignant transformation of the prostate cells. Such a phenomenon should also be reflected in a significant down-regulation of MT-encoding mRNA. Nevertheless, it is important to note that we examined only the three basic isoforms of MT1, and many other distinct isoforms have been identified^39^. Moreover, the correlation between transcription and translation is affected by a number of factors, including expression rates and protein stability^40^. Thus, single-cell analysis with RNA probes and antibodies should be performed to provide detailed insight.

Previously, it was shown that zinc biodistribution is not uniform, even in the same anatomical region of the prostate. Leitao and coworkers identified significant differences among transitional and peripheral zones^41^. However, to the best of our knowledge, the zinc-MT biodistribution in PCa is not known. Hence, we performed IMS visualization of proteins in the *m/z* range of 0–20 kDa, where MTs are expected. Instead of a number of low-intensity peaks, the three major peaks were detected, where MT isoform 1X was identified. Disparities in MT1X expression reflected the administration type, and the lowest overall intensities were achieved in sar@LIP-administered tumors, consistent with our previous electrochemical analyses. Furthermore, the administration-related changes were found mostly in the marginal regions of tumors. One plausible explanation is the enhanced angiogenesis of the margins, causing the accumulation of our designated nanotransporters. Hence, the liposomal formulations act mostly in these regions, significantly affecting prostate tumor progression. Although additional work is necessary to understand the mechanistic basis of sarcosine-induced MT down-regulation, it is apparent that pathological changes in sarcosine metabolism leading to its accumulation in cells can be an important player in prostate tumorigenesis.

In conclusion, our exploratory study revealed that sarcosine plays an important role in PCa progression. Sarcosine administration significantly affected genes linked with proliferation and apoptosis, and thus, significantly stimulated *in vivo* tumor xenograft growth. Treatment with sar@LIP decreased the amounts of tumor zinc and MT. However, the mechanistic basis of the sarcosine influence on MT regulation needs to be further investigated. In contrast, using a liposomal formulation of our developed antisarAbs, we achieved mild antitumor activity, together with positive regulation of selected pro-apoptotic genes and elevations of tumor zinc and MT. Overall, sarcosine is an important oncometabolite affecting PCa progression. We have shown that sarcosine molecules are suitable targets for the development of biological treatment modalities. Although our antibodies exhibited only moderate antitumor effects, further co-administration with various anticancer drugs should be tested to enhance the treatment efficiency. One option could be exploitation of specific inhibitors of enzymes involved in sarcosine biosynthesis, such as siRNA or aptamers. Song and coworkers have shown that siRNA knock-down of GNMT in PC-3 and LNCaP cells resulted in apparent decrease in proliferation with simultaneous increase of apoptotic cell counts^42^, however the knock-down effect on sarcosine amount was not investigated. Hence a question, whether the antiproliferative effects were due to a decrease in concentrations of intracellular sarcosine remains unanswered and further research is required to shed light into this phenomenon.

## Materials and Methods

### Chemicals

All reagents for syntheses, standards, and other chemicals were purchased from Sigma-Aldrich (St. Louis, MO, USA) in ACS purity, unless otherwise noted.

### Liposome synthesis and sarcosine and antisarAbs encapsulation

Liposomes were prepared following the lipidic thin-film hydration method. Briefly, 100 mg of cholesterol, 100 mg of 1,2-dioleoyl-sn-glycero-3-phosphoethanolamine (DOPE) and 100 mg of phosphatidylcholine were dissolved in chloroform (4.5 mL). A lipidic film was obtained by rotary evaporation of solvent. The residual chloroform was blown out with nitrogen until a thin film was formed. For encapsulation, 500 μL of sarcosine (200 μg/mL) and antisarAbs (500 μg/mL), produced in leghorn hens as described in our previous study^11^, was utilized. After addition to the lipidic film (10 mg), the samples were sonicated in an ultrasonic bath (15 min). The sample was then heated on a Biosan Thermo Shaker TS-100C (Biosan, Riga, Latvia) for 15 min at 60 °C. After cooling, FA was poured, followed by the addition of 1,1′-carbonyldiimidazole (CDI) (the molar ratio of CDI to FA was 1:1). The samples were shaken on a thermoshaker for 2 h. Finally, the liposomes were filtered using Amicon^®^ Ultra 0.5 mL centrifugal filters (Merck Millipore, Billerica, MA, USA) to remove unbound residual impurities.

### Transmission electron microscopy (TEM)

Liposomes were suspended within a drop of MilliQ purified H_2_O. The resulting suspension was covered with a grid-coated formvar film (Sigma-Aldrich) and carbon (Agar Scientific, Essex, UK). After drying, 2% ammonium molybdate was placed onto the grid, and the excess was dried. Liposomes were examined using a Philips 208 S Morgagni transmission electron microscope (FEI, Brno, Czech Republic) at 18,000× magnification and an accelerating voltage of 80 kV.

### Determination of the mean hydrodynamic diameter of the liposomal transporter

The liposome hydrodynamic diameters were examined using a Particle Size Analyzer (Zetasizer Nano ZS90, Malvern Instruments, Malvern, UK). Particles were dispersed in phosphate-buffered saline (PBS, 137 mM NaCl, 2.7 mM KCl, 1.4 mM NaH_2_PO_4_, and 4.3 mM Na_2_HPO_4_, pH 7.4) and were incubated at 25 °C for 15 min before the measurement.

### Optical characterization of liposomes

Absorbance and photoluminescence were examined using a Tecan Infinite 200 PRO multifunctional microplate reader (Tecan, Männedorf, Switzerland) in Costar^®^ UV-transparent 96-well microplates with flat bottoms (Corning Inc., Corning, NY, USA). To determine the surface modification, λ_exc_ = 360 nm of FA was used.

### Determination of loading efficiency in the liposomes

Loading efficiency (LE%) was determined by measuring the concentration of unencapsulated free sarcosine and antisarAbs in aqueous medium. For this purpose, 400 μL of liposomes was centrifuged (10,000 rpm, 15 min, 4 °C) in Amicon^®^ Ultra 0.5 mL centrifugal filters (Merck Millipore), and the concentration of free sarcosine was quantified using ion-exchange chromatography according to Heger *et al.*^6^. The concentration of free antisarAbs was examined using the Bradford assay^43^. LE% was estimated with the following formula: (Cargo_total_ − Cargo_supernatant_)/Cargo_total_ × 100.

### Cells

The PC-3 human cell line, established from a grade 4 androgen-independent prostatic adenocarcinoma, was purchased from the Health Protection Agency Culture Collection (Salisbury, UK). The cells were grown in Ham’s 12 medium with 7% fetal bovine serum (FBS) supplemented with penicillin (100 U/mL) and streptomycin (0.1 mg/mL). The PNT1A human cell line established by the immortalization of normal adult prostatic epithelial cells by transfection with a plasmid containing the SV40 genome with a defective replication origin was employed as a control model for microarray analyses (S1). The cells were cultured in RPMI-1640 medium with 10% FBS. The cells were maintained at 37 °C in a humidified incubator (Sanyo, Moriguchi, Japan) with 5% CO_2_.

### Determination of intracellular uptake of antisarAbs@LIP

To determine the intracellular uptake, antisarAbs were fluorescently labeled with CdTe quantum dots (QDs) through a synthetic heptapeptide HWR linker prior to encapsulation, as described in our previously published studies^11^,^44^. PC-3 cells were seeded onto a plate at a density of 2.0 × 10^5 ^cells/well and were incubated for 24 h. Microscopy studies were performed using an Olympus IX 71S8F-3 inverted fluorescence microscope (Olympus, Tokyo, Japan) equipped with an HBO 50 W mercury arc lamp (Osram GmbH, Munich, Germany). The 545 and 580 nm excitation filters and the 610 nm emission filter were utilized. Images were acquired with an Olympus DP73 camera and processed using Stream Basic 1.7 software (Olympus) at a resolution of 1,600 × 1,200 pixels. To evaluate the competitive inhibitory effects of FA, various FA concentrations (final FA concentrations 2; 8 and 20 mM) were utilized for pre-treatment, followed by subsequent treatment with 10 μM QD-labeled antisarAbs@LIP and incubation for 4 h. The cells were then washed with medium, trypsinized and harvested. The median QD fluorescence values were analyzed using a Tecan Infinite 200 PRO multifunctional microplate reader (Tecan, Maennedorf, Switzerland) using λ_ex_ = 500 nm and λ_em_ = 610 nm.

### Prostate tumor xenograft models

Five-week-old male nude athymic BALB/c nu/nu mice were used for xenograft studies. PC-3 cells (5 × 10^6^) were resuspended in 100 μL of PBS with 20% Matrigel (BD Biosciences, Franklin Lakes, NJ, USA) and were then implanted subcutaneously into the left flank regions of the mice under general anesthesia (1% Narkamon + 2% Rometar, 0.5 mL/100 g of weight). Growth in tumor volume was recorded using digital calipers, and tumor volumes were calculated using the formula: (*π*/6) × (*L* × *W*^2^), where *L* is the length of tumor and *W* is the width. The use of the animals followed the European Community Guidelines as accepted principles for the use of experimental animals. The experiments were performed with the approval of the Ethics Commission at the Faculty of Medicine, Masaryk University, Brno, Czech Republic.

### RNA isolation and reverse transcription

Tumors were crushed using a mortar and pestle under liquid nitrogen and were then mixed with 350 μL of Tissue Lysis Buffer (Roche, Basel, Switzerland) in an Eppendorf tube. After 30 min incubation at 25 °C, samples were centrifuged (13,000 × g at 20 °C for 2 min) using an Eppendorf 5402 microcentrifuge (Eppendorf, Hamburg, Germany). Next, 350 μL of the lysate supernatant was pipetted into the sample tube, a component of the MagNA Pure Compact RNA Isolation Kit (Roche, Basel, Switzerland). The isolation steps were performed according to the manufacturer’s instructions. The concentration of the RNA was quantified using an Infinite M200 PRO (Tecan). The RNA was converted to cDNA using a PrimeScript One Step RT-PCR Kit (TaKaRa, Mountain View, CA, USA) with the following reaction profile: 25 °C for 10 min, 37 °C for 120 min and 85 °C for 5 min. The cDNA integrity was tested by PCR of *β-actin* with the following primers: forward 5′-CCTGAACCCTAAGGCCAACC-3′ and reverse 5′-GCAATGCCTGGGTACATGGT-3′. The resulting DNA fragment (600 bp) was visualized using agarose gel electrophoresis with ethidium bromide staining as previously described by Nejdl *et al.*^45^.

### Electrochemical microarray analyses

Obtained cDNA was biotinylated on its 3′ end using the Biotin 3′ End DNA Labeling Kit (Thermo Scientific, Waltham, MA, USA) following the manufacturer’s instructions. The microarray was performed as previously described by Roth *et al.*^46^. For hybridization, Human Cancer 3711 ElectraSense 4 × 2k array slides with 1,609 DNA probes (Custom Array, Bothell, WA, USA) were first pre-hybridized for 30 min at 50 °C using 6× SSPE (0.9 M NaCl, 60 mM sodium phosphate, 6 mM EDTA), 5× Denhardt’s solution and sonicated salmon sperm DNA (100 μg/mL). Then, hybridization of biotin-labeled cDNA was performed at 50 °C for 18 h in 6× SSPE and salmon sperm DNA (100 μg/mL). Array chips were rinsed with low ionic strength 3× SSPET (3× SSPE, 0.05% Tween-20) and PBST (2× phosphate-buffered saline, pH 7.4, 0.1% Tween-20) to remove weakly bound DNA. Subsequently, array chips were blocked with biotin blocking solution for 15 min. Chips were then incubated for 30 min with poly-horseradish peroxidase-streptavidin (1:1,000 in PBS containing 1% BSA and 0.05% Tween-20). Next, chips were rinsed three times with biotin wash solution and 3,3′,5,5′-tetramethylbenzidine (TMB) rinse solution, followed by incubation with TMB substrate. Measurements were performed using the ElectraSense detection kit (Custom Array). All post-hybridization processing steps were performed at 25 °C. The expression levels of selected genes were validated through qRT-PCR using SYBR^®^ Green I Nucleic Acid Gel Stain (Invitrogen, Waltham, MA, USA) and the primers shown in Supplementary Table 1.

### Western blot analysis

Tumor tissue for detection of caspase-3, cleaved caspase-3 and β-actin was processed and analyzed according to previously published protocol^47^. In brief, after the lysis, 50 μg of proteins were separated by 12% (*w/v*) sodium dodecyl sulfate-polyacrylamide gel electrophoresis. After transfer onto polyvinylidene fluoride (PVDF) membranes and blocking with 5% (*w/v*) skim milk, the primary antibodies (Cell Signaling Technology, Danvers, MA, USA) were added overnight on the shaker at 4 °C. Then PVDF membranes were incubated with horseradish peroxidase-conjugated secondary antibodies for 1 h at 25 °C. The detection was carried out using 3-aminoethyl-9-carbazole in 0.5 M acetate buffer as substrate.

### Histological procedures

The samples were fixed in formaldehyde (10%) overnight, subsequently dehydrated in serial ethanol concentrations and embedded in paraffin wax. Sections were cut at 5 μm, mounted on glass slides, deparaffinized and stained with hematoxylin-eosin. The microscopic observations were conducted using an Olympus IX 71S8F-3 (Olympus, Tokyo, Japan).

### Matrix-assisted laser desorption/ionization time-of-flight (MALDI-TOF) imaging mass spectrometry (IMS) and peptide mass fingerprinting (PMF)

Sections of excised tumors (thickness 10 μm) were mounted onto ITO glass slides (Bruker Daltonik, GmbH). Deparaffinization and antigen retrieval were performed according to Casadonte and Caprioli^48^. The glass slides were scanned with an Epson Perfection V500 (Epson Europe, Amsterdam, Netherland) with a resolution of 2,400 DPI. Subsequently, 2,5-dihydroxybenzoic acid (DHB) (30 mg/mL in 50% methanol and 0.2% trifluoroacetic acid) was sprayed using an ImagePrep vibrational sprayer system (Bruker Daltonik, GmbH). The experiments were performed on a MALDI-TOF/TOF Bruker ultrafleXtreme (Bruker Daltonik, GmbH). The scanning raster was set to 100 μm. Analyses were performed in reflectron positive mode with a laser power of 65%. The mass spectra were typically acquired in the *m/z* range 0.5–20 kDa by averaging 1,600 sub spectra from a total of 1,600 laser shots per raster spot. After analyses, the mass spectra were automatically loaded into flexAnalysis and processed (baseline subtraction). Pseudo-colored images of spatial distribution were generated using GIMP 2.8 (http://www.gimp.org/). Identification of proteins was performed using PMF with *in situ* trypsin digestion. First, trypsin (0.05 μg/μL in water) was deposited to cover the entire tissue surface. Subsequently, DHB (30 mg/mL in 50% methanol and 0.2% trifluoroacetic acid) was deposited using a vibrational sprayer system (Bruker Daltonik, GmbH). Identification of trypsinized proteins was achieved with conventional algorithms using the MASCOT server (Matrix Science, Boston, MA, USA). The following parameters were used for database searches: trypsin was used as the enzyme, zero or one missed cleavage was allowed, taxonomy was set to *Homo sapiens*, oxidation of methionine or/and *N*-term acetylation were added as variable modifications, peptide tolerance was set to ±0.5 Da, and mass values were set as MH^+^.

### Preparation of tumor tissue to determine zinc content

Ten milligrams of tumor was digested in nitric acid (65% *v/v*) and hydrogen peroxide (30 *v/v*) at a ratio of 7:3 in a Microwave System 3000 (Anton Paar GmbH, Graz, Austria) using a MG-65 rotor. Microwave power was set to 100 W (30 min) at 140 °C.

### Atomic absorption spectrometry of zinc

Measurements were performed on a 280Z atomic absorption spectrometer (Agilent, Technologies, Santa Clara, CA, USA) with electrothermal atomization and Zeeman background correction. Zinc was measured on the primary wavelength Zn 213.9 nm (spectral bandwidth 0.5 nm, lamp current 10 mA) in the presence of a Pd chemical modifier.

### Preparation of tumor tissue to determine total protein and MT amounts

Tumors were frozen with liquid nitrogen and homogenized using a SONOPLUS mini20 ultrasonic homogenizer (Bandelin Electronic, Berlin, Germany). Subsequently, 1 mL of 0.2 M phosphate buffer (pH, 7.0) was added, and the sample was homogenized for 5 min. The homogenates were further centrifuged using a Microcentrifuge 5417R (Eppendorf, Hamburg, Germany) at 15,000 × *g*, 4 °C for 15 min. Finally, the supernatant was filtered through a membrane filter (0.45 μm nylon filter disk; Millipore, Billerica, MA, USA) and analyzed.

### Determination of total protein content

The total protein concentrations were utilized for results normalization and were quantified using a SKALAB CBT 600T kit (Skalab, Svitavy, Czech Republic) according to manufacturer instructions with a BS-400 automated spectrophotometer (Mindray, Schenzhen, China).

### Determination of amount of total MTs

MT was quantified using differential pulse voltammetry (747 VA Stand, connected to a 693 VA processor and 695 Autosampler, Metrohm, Herissau, Switzerland) with the conditions used in our previous study^49^.

### Descriptive statistics

Results are expressed as the mean ± standard deviation unless noted otherwise. Differences between groups were analyzed using the paired *t*-test and analysis of variance (ANOVA). Unless noted otherwise, the threshold for significance was *p* < 0.05. Statistica 12 software (StatSoft, Tulsa, OK, USA) was employed for analyses.

## Additional Information

**How to cite this article**: Heger, Z. *et al.* Prostate tumor attenuation in the nu/nu murine model due to anti-sarcosine antibodies in folate-targeted liposomes. *Sci. Rep.* **6**, 33379; doi: 10.1038/srep33379 (2016).

## Supplementary Material

Supplementary Information

## Figures and Tables

**Table 1:** Loading efficiencies (LE%) of sarcosine and antisarAbs in folate-targeted liposomes.

Carrier	Cargo	LE%	SD
FA- Liposomes	Sarcosine	78.9	6.21
	antisarAbs	56.2	5.16

Values are expressed as the mean of three independent replicates (*n* = 3).
