# Supplementary material for: Prostate tumor attenuation in the nu/nu murine model due to anti-sarcosine antibodies in folate-targeted liposomes
Source: Sci Rep. 2016 Sep 20;6:33379. doi: 10.1038/srep33379 (PMC5028781; doi:10.1038/srep33379)
Supplement: Supplementary Information [file srep33379-s1.pdf]

Prostate tumor attenuation in the nu/nu murine model due to anti-sarcosine antibodies in folate-targeted liposomes

Zbynek Heger[1,2], Hana Polanska[3], Miguel Angel Merlos Rodrigo[1,2], Roman Guran[1,2], Pavel Kulich[4], Pavel Kopel[1,2], Michal Masarik[3], Tomas Eckschlager[5], Marie Stiborova[6], Rene Kizek[1,2], Vojtech Adam[1,2*]

[1]*Central European Institute of Technology, Brno University of Technology, Purkynova 123, CZ-612 00 Brno, Czech Republic - European Union*

[2]*Department of Chemistry and Biochemistry, Mendel University in Brno, Zemedelska 1, CZ 613 00 Brno, Czech Republic - European Union*

[3]*Department of Pathological Physiology, Faculty of Medicine, Masaryk University, Kamenice 5, Brno CZ-625 00, Czech Republic - European Union*

[4]*Department of Chemistry and Toxicology, Veterinary Research Institute, Hudcova 296/70, CZ 621 00 Brno, Czech Republic - European Union*

[5]*Department of Pediatric Hematology and Oncology, 2nd Faculty of Medicine, Charles University, and University Hospital Motol, V Uvalu 84, CZ-150 06 Prague 5, Czech Republic - European Union*

[6]*Department of Biochemistry, Faculty of Science, Charles University, Albertov 2030, CZ-128 40 Prague 2, Czech Republic - European Union*

***Corresponding author**

Vojtech Adam, Department of Chemistry and Biochemistry, Mendel University in Brno, Zemedelska 1, CZ-613 00 Brno, Czech Republic; E-mail: vojtech.adam@mendelu.cz; Phone: +420-5-4513-3350; Fax: +420-5-4521-2044

Table S1: List of primers employed for validation of selected microarray results using qRT-PCR

Gene	Symbol	Primer pair (5´-3´)*	T_m
ARP2 actin-related protein 2 homolog	*ACTR2*	ATCACGGTTGGAACGAGAAC<br>CACACGGACACCCTTTTCTT	60.0
DnaJ (Hsp40) homolog, subfamily B, member 6	*DNAJB6*	AAGCAAGTAGCGGAGGCATA<br>AATGAAAATGGGTCCCTTCC	60.0
Kallikrein 3, (prostate specific antigen) PSA	*KLK3*	TTGTCTTCCTCACCCTGTCC<br>AGTCATCACCTGGCTTCCTG	60.0
Speckle-type POZ protein	*SPOP*	GAAGTTCGGGCAAAATTCAA<br>CAGTCTTTGCCTTGCACAAA	60.0
Procollagen C-endopeptidase enhancer	*PCOLCE*	AGGCTTCCTGCTCTGGTACA<br>GCTCCAGGTCAAACTTCTCG	60.0
Myeloid/lymphoid or mixed-lineage leukemia; translocated to 10	*MLLT10*	AGAGGCAGTGGAGTGAAGGA<br>GGAAAAGGCACTGAAAGCTG	60.0
CSE1 chromosome segregation 1-like	*CSE1L*	CGATACCTGCCTCGTTTTGT<br>GCAGCCCTGCGTCTAGTATC	60.0
Gamma-aminobutyric acid (GABA) B receptor, 1	*GABBR1*	CCTCATCACTGCTCCTGTCA<br>CCCTGTCTTCATGGTGTCCT	59.9
Glutaminase	*GLS*	TTCTCAGGGCAGTTTGCTTT<br>TTGCCCATCTTATCCAGAGG	59.9
Ubiquitin-conjugating enzyme E2C	*UBE2C*	CTCGCTAGAGTTCCCCAGTG<br>AGGGCAGACCACTTTTCCTT	60.0
Solute carrier family 6, member 3	*SLC6A3*	ACCTTCCTCCTGTCCCTGTT<br>CACCATAGAACCAGGCCACT	60.0
Hypothetical protein MAC30	*MAC30*	TTCTGTTTTGCGAGCTTGTG<br>GAAACCACTGGCTTTGGAGA	60.0
Carcinoembryonic antigen-related cell adhesion molecule 1	*CEACAM1*	ACGTCACCCAGAATGACACA<br>CTCCACAGGGTTGGAGTTGT	60.0
Colony stimulating factor 2 receptor, alpha, low affinity	*CSF2RA*	AGCCCAGAGCAAAACACAGT<br>CTCGTCTTCCACCTCATGGT	59.9
Tumor necrosis factor receptor superfamily, member 8	*TNFRSF8*	ATGGCTGAGAAGGACACCAC<br>ACACCAGGATCACCCAGAAG	60.0

*Upper and lower sequences represent forward and reverse primers, respectively; T_m is melting temperature of specific product.

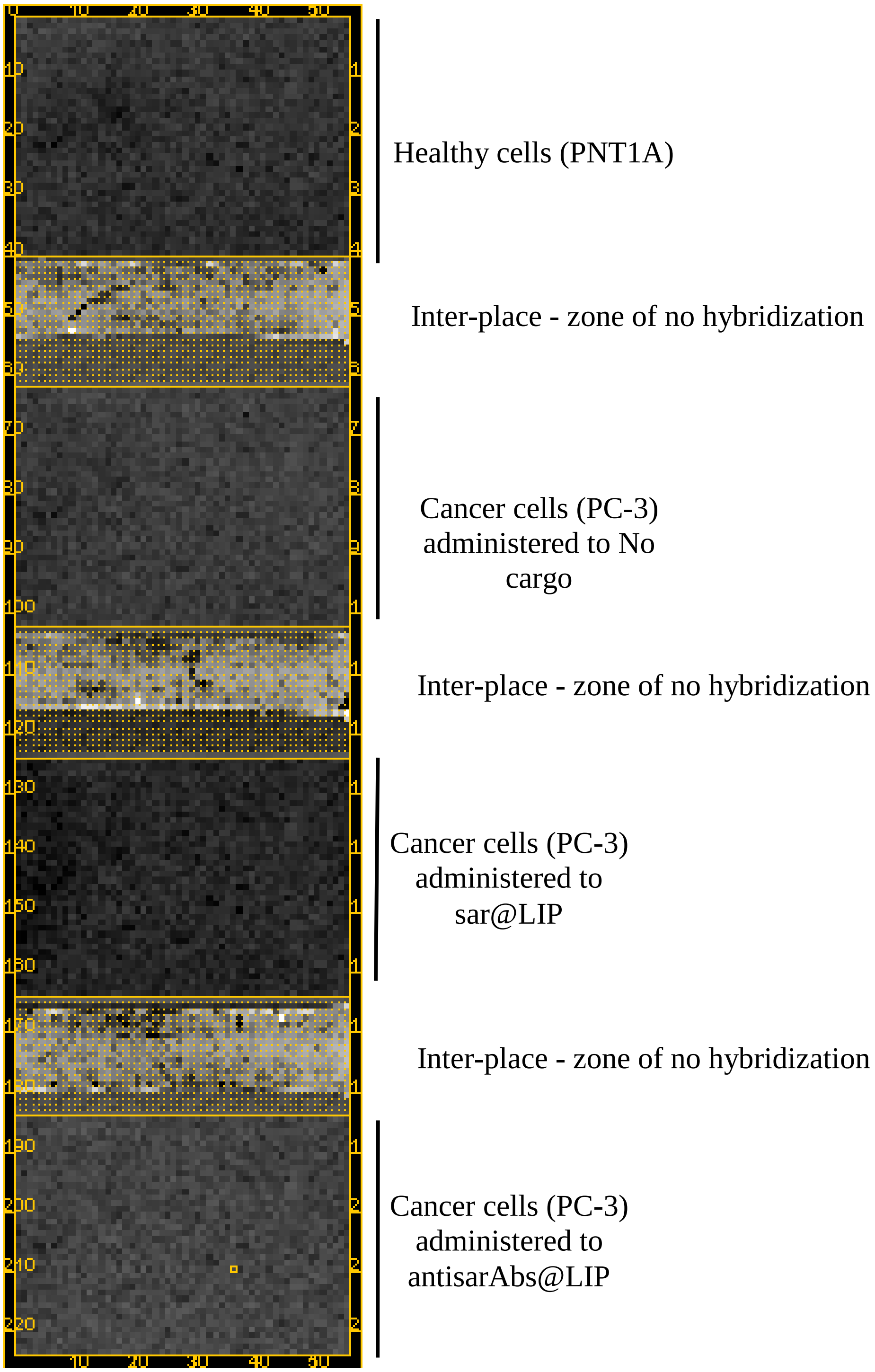

Figure S1: Microarray heatmap. Each spot on heatmap shows expression of one gene. Gray scale intensity represents the rate of individual mRNA expression.

Table S2: List of genes up- or down-regulated after treatment with AntisarAbs@LIP

**Up-regulated**				
**Gene**	**Symbol**	**NCBI database**	**Fold ratio**	**SD (*n* = 3)**
Tumor necrosis factor receptor superfamily, member 8	*TNFRSF8*	NM_001243	2.9	1.6
Proliferating cell nuclear antigen	*PCNA*	NM_002592	2.7	1.6
DnaJ (Hsp40) homolog, subfamily B, member 6	*DNAJB6*	NM_005494	2.6	1.0
Insulin-like growth factor binding protein 3	*IGFBP3*	NM_000598	2.5	0.9
Fc fragment of IgG, low affinity IIIa, receptor (CD16a)	*FCGR3A*	NM_000570	2.3	0.9
Kallikrein 3, (prostate specific antigen) PSA	*KLK3*	NM_001030048	2.3	0.9
Interleukin 11	*IL11*	NM_000641	2.3	0.9
Chromodomain helicase DNA binding protein 1	*CHD1*	NM_001270	2.3	0.5
Phosphatidylserine synthase 1	*PTDSS1*	NM_014754	2.2	0.6
Metadherin	*LYRIC*	NM_178812	2.1	0.4
Solute carrier family 6, member 3	*SLC7A1*	NM_003045	2.1	0.6
AE binding protein 1	*AEBP1*	NM_001129	2.0	0.3
Guanylate binding protein 1, interferon inducible	*GBP1*	NM_001551	1.9	0.2
CD53 antigen	*CD53*	NM_000560	1.9	0.3
Thymidine kinase 1, soluble	*TK1*	NM_003258	1.9	0.4
Desmoglein 3 (pemphigus vulgaris antigen)	*DSG3*	NM_001944	1.9	0.3
Protein kinase, cAMP-dependent, regulatory, type II, beta	*PRKAR2B*	NM_002736	1.9	0.3
Chemokine (C-X-C-motif) ligand 3	*CXCL3*	NM_002090	1.9	0.3
Carbonic anhydrase IX	*CA9*	NM_001216	1.9	0.3
Hypothetical protein MAC30	*MAC30*	NM_014573	1.9	0.9
Proprotein convertase subtilisin/kexin type 1	*PCSK1*	NM_000439	1.9	0.3
Progesterone receptor membrane component 1	*PGRMC1*	NM_006667	1.8	0.3
Choline kinase alpha	*CHKA*	NM_001277	1.8	0.3
Melanoma antigen family A, 3	*MAGEA3*	NM_005362	1.8	0.2
Interleukin 6 (interferon, beta 2)	*IL6*	NM_005362	1.8	0.4
Phosphofructokinase, platelet	*PFKP*	NM_002627	1.8	0.2
Topoisomerase (DNA) II alpha 170kDa	*TOP2A*	NM_002627	1.8	0.4
Bone marrow stromal cell antigen 2	*BST2*	NM_004335	1.8	0.5
Caspase 4, apoptosis-related cysteine peptidase	*CASP4*	NM_033307	1.8	0.3
Actin-like 6A	*ACTL6A*	NM_004301	1.8	0.3

Chemokine (C-X-C motif) receptor 4	*CXCR4*	NM_001008540	1.8	0.2
Testis enhanced gene transcript (BAX inhibitor 1)	*TEGT*	NM_003217	1.8	0.2
G1 to S phase transition 1	*GSPT1*	NM_002094	1.7	0.2
Platelet-derived growth factor receptor, beta polypeptide	*PDGFRB*	NM_002609	1.7	0.2
Integrin, alpha M (complement component receptor 3, alpha)	*ITGAM*	NM_000632	1.7	0.1
Cathepsin L2	*CTSL2*	NM_001333	1.7	0.2
Tropomyosin 2 (beta)	*TPM2*	NM_003289	1.7	0.2
Alcohol dehydrogenase 1A (class I), alpha polypeptide	*ADH1A*	NM_000667	1.7	0.1
Bcl2-associated X protein	*BAX*	NM_000435	1.7	0.1
Trefoil factor 3	*TFF3*	NM_003226	1.7	0.3
Solute carrier family 25, member 6	*SLC25A4*	NM_001151	1.7	0.3
Metallothionein M	*MTM*	NM_175620	1.7	0.1
Dynein, cytoplasmic, heavy polypeptide 1	*DNCH1*	NM_001376	1.7	0.4
L-3-hydroxyacyl-Coenzyme A dehydrogenase, short chain	*HADHSC*	NM_005327	1.7	0.2
Basic leucine zipper transcription factor, ATF-like	*BATF*	NM_006399	1.7	0.2
Phosphoglycerate mutase 1	*PGAM1*	NM_002629	1.7	0.1
Surfactant, pulmonary-associated protein A1	*SFTPA1*	NM_005411	1.7	0.2
Eukaryotic translation elongation factor 1 beta 2	*EEF1B2*	NM_021121	1.7	0.1
Transforming growth factor, beta-induced, 68kDa	*TGFB1*	NM_000358	1.7	0.1
Protease, serine, 8 (prostasin)	*PRSS8*	NM_002773	1.7	0.2
Phosphoglycerate kinase 1	*PGK1*	NM_000291	1.6	0.1
ARP2 actin-related protein 2 homolog	*ACTR2*	NM_001005386	1.6	0.3
Cold inducible RNA binding protein	*CIRBP*	NM_001280	1.6	0.1
NIMA (never in mitosis gene a)-related kinase 2	*NEK2*	NM_002497	1.6	0.1
Mucin 19	*MUC19*	NM_495921	1.6	0.1
CD24 antigen	*CD24*	NM_013230	1.6	0.2
ATPase family, AAA domain containing 2	*ATAD2*	NM_014109	1.6	0.3
Arrestin, beta 2	*ARRB2*	NM_004313	1.6	0.2
V-erb-b2 erythroblastic leukemia viral oncogene homolog 3	*ERBB3*	NM_001005915	1.6	0.2
Glutathione peroxidase 1	*GPX1*	NM_201397	1.6	0.2
Fms-related tyrosine kinase 1	*FLT1*	NM_002019	1.6	0.2
Solute carrier family 16, member	*SLC16A3*	NM_004207	1.6	0.12

3				
Interleukin 8	*IL8*	NM_000584	1.6	0.4
ATPase, H+ transporting, lysosomal 42kDa, V1 subunit C, isoform 1	*ATP6V1C1*	NM_001007254	1.6	0.1
HCLS1 associated protein X-1	*HAX1*	NM_001018837	1.6	0.2
Chloride channel, nucleotide-sensitive, 1A	*CLNS1A*	NM_001293	1.6	0.2
Thyroid hormone receptor, alpha (erythroblastic leukemia viral)	*THRA*	NM_003250	1.6	0.1
Calponin 1, basic, smooth muscle	*CNN1*	NM_001299	1.6	0.4
Programmed cell death 2	*PDCD2*	NM_002598	1.6	0.2
Sialidase 1 (lysosomal sialidase)	*NEU1*	NM_000434	1.6	0.1
Solute carrier family 6, member 3	*SLC6A3*	NM_001044	1.6	0.2
Caudal type homeo box transcription factor 1	*CDX1*	NM_001804	1.6	0.5
Quinoid dihydropteridine reductase	*QDPR*	NM_000320	1.6	0.5
KIAA0020	*KIAA0020*	NM_001031691	1.6	0.3
Eukaryotic translation elongation factor 1 delta	*EEF1D*	NM_001960	1.6	0.2
ADP-ribosylation-like factor 6 interacting protein 5	*ARL6IP5*	NM_006407	1.6	0.1
Cystatin E/M	*CST6*	NM_001323	1.6	0.1
Coagulation factor II (thrombin) receptor	*F2R*	NM_001323	1.6	0.2
Transketolase (Wernicke-Korsakoff syndrome)	*TKT*	NM_001054	1.6	0.3
Phosphoribosyl pyrophosphate synthetase 2	*PRPS2*	NM_002765	1.6	0.2
Thrombospondin 2	*THBS2*	NM_003247	1.6	0.1
Ubiquitin-conjugating enzyme E2I	*UBE2I*	NM_003345	1.6	0.3
Claudin 3	*CLDN3*	NM_001306	1.6	0.1
3-oxoacid CoA transferase 1	*OXCT1*	NM_000436	1.6	0.3
Nuclear receptor subfamily 4, group A, member 1	*NR4A1*	NM_173158	1.6	0.1
Ornithine decarboxylase 1	*ODC1*	NM_002539	1.6	0.1
Phosphoenolpyruvate carboxykinase 1 (soluble)	*PCK1*	NM_002591	1.6	0.1
General transcription factor IIIA	*GTF3A*	NM_002097	1.6	0.1
Chloride intracellular channel 1	*CLC1*	NM_001288	1.6	0.2
Guanine nucleotide binding protein (G protein), alpha z polypeptide	*GNAZ*	NM_002073	1.6	0.2
Effector cell peptidase receptor 1	*BIRC5*	NM_001012270	1.6	0.1
Speckle-type POZ protein	*SPOP*	NM_001007226	1.6	0.1
Solute carrier family 1, member 3	*SLC1A3*	NM_004172	1.6	0.1

TAP binding protein (tapasin)	*TAPBP*	NM_172208	1.6	0.3
Poly(A) binding protein, cytoplasmic 1	*PABPC1*	NM_002568	1.6	0.2
Amino-terminal enhancer of split	*AES*	NM_001130	1.6	0.1
Keratin 20	*KRT20*	NM_019010	1.6	0.1
Interferon induced transmembrane protein 2 (1-8D)	*IFITM2*	NM_006435	1.5	0.2
Fructose-1,6-bisphosphatase 1	*FBP1*	NM_000507	1.5	0.2
Insulin-like growth factor binding protein 5	*IGFBP5*	NM_000599	1.5	0.1
Major histocompatibility complex, class II, DQ alpha 1	*HLA-DQA1*	NM_002122	1.5	0.1
Metallothionein 1X	*MT1X*	NM_005952	1.5	0.1
Kinesin family member 14	*KIF14*	NM_014875	1.5	0.1
Zinc finger protein 161	*ZNF161*	NM_007146	1.5	0.1
Phosphoinositide-3-kinase, class 2, beta polypeptide	*PIK3C2B*	NM_002646	1.5	0.1
Procollagen C-endopeptidase enhancer	*PCOLCE*	NM_002593	1.5	0.0
Myeloid/lymphoid or mixed-lineage leukemia	*MLLT10*	NM_001009569	1.5	0.0
IGF-II mRNA-binding protein 3	*IMP-3*	NM_006547	1.5	0.2
Chemokine (C-X-C motif) ligand 11	*CXCL11*	NM_005409	1.5	0.1
Topoisomerase (DNA) I	*TOP1*	NM_003286	1.5	0.1
Neutrophil cytosolic factor 2	*NCF2*	NM_000433	1.5	0.1
Protein kinase C, iota	*PRKCI*	NM_002740	1.5	0.1
Disabled homolog 2, mitogen-responsive phosphoprotein	*DAB2*	NM_138709	1.5	0.2
Serpin peptidase inhibitor, clade E, member 2	*SERPINE2*	NM_006216	1.5	0.1
Cytochrome P450, family 2, subfamily A, polypeptide 6	*CYP2A6*	NM_000762	1.5	0.1
Transcobalamin I (vitamin B12 binding protein, R binder family)	*TCN1*	NM_001062	1.5	0.1
Rho GDP dissociation inhibitor (GDI) beta	*ARHGDIB*	NM_001175	1.5	0.2
Phosphoglucomutase 1	*PGM1*	NM_002633	1.5	0.0
Protein kinase, cAMP-dependent, catalytic, beta	*PRKACB*	NM_207578	1.5	0.2
Signal sequence receptor, beta	*SSR2*	NM_003145	1.5	0.1
RAN binding protein 9	*RANBP9*	NM_005493	1.5	0.1
Peroxisomal biogenesis factor 12	*PEX12*	NM_000286	1.5	0.1
Collagen, type II, alpha 1	*COL2A1*	NM_001844	1.5	0.2
Phospholamban	*PLN*	NM_002667	1.5	0.1
Mal, T-cell differentiation protein-like	*BENE*	NM_003145	1.5	0.1
Neuroligin 2	*NLGN2*	NM_004576	1.5	0.1
Cytochrome c oxidase subunit Va	*COX5A*	NM_005434	1.5	0.1

Protein phosphatase 2, regulatory subunit B (PR 52), beta isoform	*PPP2R2B*	NM_004358	1.5	0.1
Mal, T-cell differentiation protein-like	*BENE*	NM_000089	1.5	0.1
Cell division cycle 25B	*CDC25B*	NM_000224	1.5	0.1
Collagen, type I, alpha 2	*COL1A2*	NM_002055	1.5	0.1
Keratin 18	*KRT18*	NM_181800	1.5	0.2
Glial fibrillary acidic protein	*GFAB*	NM_001949	1.5	0.1
Ubiquitin-conjugating enzyme E2C	*UBEC2C*	NM_003145	1.5	0.1
Preferentially expressed antigen in melanoma	*PRAME*	NM_006115	1.5	0.1
**Down-regulated**				
LUC7-like	*LUC7L*	NM_201412	0.5	0.1
Dystonin	*DST*	NM_000112	0.5	0.1
Gamma-aminobutyric acid (GABA) B receptor, 1	*GABBR1*	NM_001470	0.5	0.0
Polymerase I and transcript release factor	*PTRF*	NM_012232	0.4	0.1
Retinol binding protein 3, interstitial	*RBP3*	NM_002900	0.4	0.0
CD164 antigen, sialomucin	*CD164*	NM_006016	0.3	0.2
KIAA1217	*KIAA1217*	NM_019590	0.3	0.1
MAD2 mitotic arrest deficient-like 1 (yeast)	*MAD2L1*	NM_002358	0.3	0.2
Myosin, heavy polypeptide 11, smooth muscle	*MYH11*	NM_002474	0.1	0.1
Anaphase promoting complex subunit 5	*ANAPC5*	NM_016237	0.1	0.3

Table S3: List of genes up- or down-regulated after treatment with Sar@LIP

**Up-regulated**				
**Gene**	**Symbol**	**NCBI database**	**Fold ratio**	**SD (*n* = 3)**
Bromodomain containing 4	*BRD4*	NM_058243	4.1	0.7
Colony stimulating factor 2 receptor, alpha, low-affinity	*CSF2RA*	NM_172247	2.7	0.3
Thymidylate synthetase	*TYMS*	NM_001071	2.4	0.7
Heterogeneous nuclear ribonucleoprotein A1	*HNRPA1*	NM_002136	2.4	0.3
Mitogen-activated protein kinase 12	*IVNS1ABP*	NM_006469	2.3	0.2
Kallikrein 4 (prostase, enamel matrix, prostate)	*KLK4*	NM_004917	2.2	0.1
Myeloid/lymphoid or mixed-lineage leukemia)	*MLLT10*	NM_001009569	2.2	0.0
Gamma-aminobutyric acid (GABA) B receptor, 1	*GABBR1*	NM_001470	2.1	0.4

Kallikrein 3, (prostate specific antigen)	*KLK3*	NM_001030050	2.1	0.3
Procollagen C-endopeptidase enhancer	*PCOLCE*	NM_002593	2.0	0.0
Stanniocalcin 1	*STC1*	NM_003155	2.0	0.0
Zinc finger protein 36, C3H type, homolog (mouse)	*ZFP36*	NM_003407	2.0	0.3
Peroxiredoxin 5	*PRDX5*	NM_012094	2.0	0.0
Programmed cell death 2	*PDCD2*	NM_144781	2.0	0.0
Nuclear receptor subfamily 5, group A, member 2	*NR5A2*	NM_003822	2.0	0.0
Ribophorin II	*RPN2*	NM_002951	2.0	0.2
Chemokine (C-X-C motif) ligand 10	*CXCL10*	NM_001565	1.9	0.0
G protein-coupled receptor 126	*GPR126*	NM_001032394	1.9	0.0
V-erb-b2 erythroblastic leukemia viral oncogene homolog 3 (avian)	*ERBB3*	NM_001005915	1.9	0.1
Serine/threonine kinase 6	*STK6*	NM_003600	1.9	0.3
Immunoglobulin lambda-like polypeptide 1	*IGLL1*	NM_020070	1.9	0.1
Desmuslin	*DMN*	NM_015286	1.9	0.2
RER1 retention in endoplasmic reticulum 1 homolog	*RER1*	NM_007033	1.9	0.0
Protein regulator of cytokinesis 1	*PRC1*	NM_199413	1.9	0.0
Nidogen 2 (osteonidogen)	*NID2*	NM_007361	1.9	0.1
Prostate androgen-regulated transcript 1	*PART1*	NM_016590	1.9	0.0
Reticulocalbin 1, EF-hand calcium binding domain	*RCN1*	NM_001655	1.9	0.0
Transcription factor 7 (T-cell specific, HMG-box)	*TCF7*	NM_201633	1.9	0.5
Purine-rich element binding protein B	*PURB*	NM_033224	1.8	0.2
FBJ murine osteosarcoma viral oncogene homolog	*c-FOS*	NM_001001567	1.8	0.0
Forkhead box P1	*FOXP1*	NM_001012505	1.8	0.2
Phosphoenolpyruvate carboxykinase 1 (soluble)	*PCK1*	NM_002591	1.8	0.0
DC13 protein	*DC13*	NM_020188	1.8	0.0
Netrin 4	*NTN4*	NM_021229	1.8	0.0
Cyclin D-type binding-protein 1	*CCNDBP1*	NM_012142	1.8	0.0
Small nuclear ribonucleoprotein polypeptide N	*SNRPN*	NM_003097	1.7	0.2
NDRG family member 2	*NDRG2*	NM_016250	1.7	0.1
Endoplasmic reticulum-golgi intermediate compartment protein	*KIAA1181*	NM_020462	1.7	0.1
Fibroblast growth factor 20	*FGF20*	NM_019851	1.7	0.5
Glutaminase	*GLS*	NM_014905	1.7	0.0
Hepatocyte cell adhesion	*NDRG2*	NM_152722	1.7	0.1

molecule				
Kallikrein 10	*KLK10*	NM_002776	1.7	0.1
Procollagen-proline, 2-oxoglutarate 4-dioxygenase	*P4HB*	NM_000918	1.7	0.0
Speckle-type POZ protein	*SPOP*	NM_001007226	1.7	0.0
Nuclear receptor subfamily 2, group E, member 3	*NR2E3*	NM_016346	1.7	0.0
Jun proto-oncogene	*c-JUN*	NM_006101	1.7	0.0
Matrix metalloproteinase 2	*MMP2*	NM_004530	1.7	0.0
WAP four-disulphide core domain 2	*WFDC2*	NM_006103	1.7	0.0
Cadherin 3, type 1, P-cadherin (placental)	*CDH3*	NM_001793	1.7	0.0
Pyridoxal (pyridoxine, vitamin B6) kinase	*PDXK*	NM_003681	1.7	0.0
Melanoma cell adhesion molecule	*MCAM*	NM_006500	1.7	0.0
Exosome component 9	*EXOSC9*	NM_005033	1.7	0.0
Proliferation-associated 2G4, 38kDa	*PA2G4*	NM_006191	1.7	0.0
Cell division cycle 2-like 6 (CDK8-like)	*CDC2L6*	NM_015076	1.7	0.0
Protein tyrosine phosphatase, receptor type, O	*PTPRO*	NM_002848	1.6	0.0
Interleukin 29 (interferon, lambda 1)	*IL29*	NM_172140	1.6	0.1
ARP2 actin-related protein 2 homolog (yeast)	*ACTR2*	NM_001005386	1.6	0.4
Lactotransferrin	*LTF*	NM_002343	1.6	0.0
Ketohexokinase (fructokinase)	*KHK*	NM_000221	1.6	0.0
Cyclin D1	*CCND1*	NM_053056	1.6	0.0
Proprotein convertase subtilisin/kexin type 6	*PCSK6*	NM_138325	1.6	0.1
Stathmin 1/oncoprotein 18	*STMN1*	NM_203399	1.6	0.0
Mitogen-activated protein kinase 14	*MAPK14*	NM_139013	1.6	0.0
Alcohol dehydrogenase 1A (class I), alpha polypeptide	*ADH1A*	NM_000667	1.6	0.1
Splicing factor, arginine/serine-rich 6	*SFRS6*	NM_006275	1.6	0.0
Homeo box B5	*HOXB5*	NM_002147	1.6	0.0
ROD1 regulator of differentiation 1 (S. pombe)	*ROD1*	NM_005156	1.6	0.1
Polymerase I and transcript release factor	*PTRF*	NM_012232	1.6	0.4
Pirin (iron-binding nuclear protein)	*PIR*	NM_001018109	1.6	0.1
Cadherin 11, type 2, OB-cadherin (osteoblast)	*CDH11*	NM_001797	1.6	0.0
Prostate stem cell antigen	*PSCA*	NM_005672	1.6	0.0

Protein kinase, cAMP-dependent, catalytic, beta	*PRKACB*	NM_207578	1.6	0.0
Replication factor C (activator 1) 4, 37kDa	*RFC4*	NM_181573	1.6	0.0
CSE1 chromosome segregation 1-like	*CSE1L*	NM_001316	1.6	0.0
Latexin	*LXN*	NM_020169	1.6	0.0
Anaphase promoting complex subunit 5	*ANAPC5*	NM_016237	1.6	0.6
Dystonin	*DST*	NM_020388	1.6	0.0
Protein phosphatase 3 (formerly 2B), catalytic subunit	*PPP3CB*	NM_021132	1.6	0.0
Kallikrein 12	*KLK12*	NM_145894	1.6	0.0
Phosphoinositide-3-kinase, class 2, beta polypeptide	*PIK3C2B*	NM_002646	1.6	0.0
Adaptor-related protein complex 2, beta 1 subunit	*AP2B1*	NM_001030006	1.6	0.1
DnaJ (Hsp40) homolog, subfamily B, member 6	*DNAJB6*	NM_058246	1.6	0.8
Small nuclear ribonucleoprotein polypeptides B and B1	*SNRPB*	NM_003091	1.6	0.0
Microsomal glutathione S-transferase 3	*MGST3*	NM_004528	1.6	0.1
Cadherin 12, type 2 (N-cadherin 2)	*CDH12*	NM_004061	1.6	0.0
BTG family, member 2	*BTG2*	NM_006763	1.6	0.1
Promyelocytic leukemia	*PML*	NM_002675	1.6	0.1
Tight junction protein 3 (zona occludens 3)	*TJP3*	NM_014428	1.6	0.0
Kallikrein 13	*KLK13*	NM_015596	1.6	0.0
Solute carrier family 6 (neurotransmitter transporter, dopamine), member 3	*SLC6A3*	NM_001044	1.6	0.2
Myeloid/lymphoid or mixed-lineage leukemia	*MLLT7*	NM_005938	1.6	0.0
Lysosomal-associated membrane protein 2	*LAMP2*	NM_013995	1.6	0.1
Procollagen-proline, 2-oxoglutarate 4-dioxygenase	*P4HA1*	NM_000917	1.6	0.0
Fibroblast growth factor 13	*FGF13*	NM_004114	1.6	0.1
Heterogeneous nuclear ribonucleoprotein H3 (2H9)	*HNRPH3*	NM_012207	1.6	0.0
Pre-B-cell colony enhancing factor 1	*PBEF1*	NM_005746	1.6	0.0
Gamma-glutamyltransferase 1	*GGT1*	NM_005265	1.6	0.0
Tripartite motif-containing 37	*TRIM37*	NM_001005207	1.6	0.0
Ubiquitin-conjugating enzyme E2C	*UBE2C*	NM_181800	1.6	0.1
ADAM metallopeptidase domain	*ADAM17*	NM_003183	1.6	0.0

17				
Hypothetical protein MAC30	*MAC30*	NM_014573	1.6	0.6
Nudix (nucleoside diphosphate linked moiety X)-type motif 3	*NUDT3*	NM_006703	1.6	0.0
Quaking homolog, KH domain RNA binding (mouse)	*QKI*	NM_006775	1.6	0.0
GDP dissociation inhibitor 1	*GDI1*	NM_001493	1.6	0.0
Transferrin receptor (p90, CD71)	*TFRC*	NM_003234	1.6	0.0
Solute carrier family 43, member 1	*SLC43A1*	NM_003627	1.5	0.0
Splicing factor proline/glutamine-rich	*SFPQ*	NM_005066	1.5	0.5
Disabled homolog 2, mitogen-responsive phosphoprotein	*DAB2*	NM_138709	1.5	0.2
RAB27A, member RAS oncogene family	*RAB27A*	NM_004580	1.5	0.1
Small EDRK-rich factor 1A (telomeric)	*SERF1A*	NM_021967	1.5	0.0
ADP-ribosylation-like factor 6 interacting protein 5	*ARL6IP5*	NM_006407	1.5	0.1
CCAAT/enhancer binding protein (C/EBP), delta	*CEBPD*	NM_005195	1.5	0.0
Pitrilysin metallopeptidase 1	*PITRM1*	NM_014889	1.5	0.1
Mitogen-activated protein kinase 10	*MAPK10*	NM_138980	1.5	0.0
LUC7-like (S. cerevisiae)	*LUC7L*	NM_201412	1.5	0.1
Paralemmin 2	*PALM2-AKAP2*	NM_007203	1.5	6.1
Jerky homolog (mouse)	*JRK*	NM_003724	1.5	0.1
Epithelial membrane protein 3	*EMP3*	NM_001425	1.5	0.0
Tumor susceptibility gene 101	*TSG101*	NM_006292	1.5	0.2
Ephrin-A1	*EFNA1*	NM_004428	1.5	0.0
Golgi SNAP receptor complex member 1	*GOSR1*	NM_001007024	1.5	0.0
Ring finger protein 14	*RNF14*	NM_004290	1.5	0.0
Erythrocyte membrane protein band 4.1-like 2	*EPB41L2*	NM_001431	1.5	0.4
Plasminogen activator, tissue	*PLAT*	NM_033011	1.5	0.0
Synaptotagmin binding, cytoplasmic RNA interacting protein	*SYNCRIP*	NM_006372	1.5	0.1
DAB2 interacting protein	*DAB2IP*	NM_006372	1.5	0.1
TAP binding protein (tapasin)	*TAPBP*	NM_003190	1.5	0.3
Chemokine (C-X-C motif) ligand 9	*CXCL9*	NM_002416	1.5	0.0
Hypothetical protein FLJ20297	*FLJ20297*	NM_017751	1.5	0.0
Paternally expressed 3	*PEG3*	NM_006210	1.5	0.0
Cathepsin C	*CTSC*	NM_148170	1.5	0.0
S100 calcium binding protein A9 (calgranulin B)	*S100A9*	NM_002965	1.5	0.0

Antigen p97	*MFI2*	NM_005929	1.5	0.0
Mitogen-activated protein kinase 8	*MAPK8*	NM_015133	1.5	0.0
E2F transcription factor 3	*E2F3*	NM_005493	1.5	0.1
**Down-regulated**				
Proprotein convertase subtilisin/kexin type 6	*PCSK6*	NM_138325	0.5	0.3
Purine-rich element binding protein A	*PURA*	NM_005859	0.3	0.4
KIAA0882 protein	*KIAA0882*	NM_015130	0.2	0.4
Phosphatidylserine synthase 1	*PTDSS1*	NM_014754	0.2	0.5
GRB2-associated binding protein 2	*GAB2*	NM_012296	0.1	0.5
RNA binding protein S1, serine-rich domain	*RNPS1*	NM_006711	0.1	0.5
MAD2 mitotic arrest deficient-like 1	*MAD2L1*	NM_002358	0.1	0.7
Nuclear receptor subfamily 1, group H, member 2	*NR1H2*	NM_007121	0.1	0.5
